# A pragmatic assessment of the relative efficiency of outreach chlamydia screening events conducted in non-clinical settings

**DOI:** 10.1186/1471-2458-12-341

**Published:** 2012-05-09

**Authors:** Francis J Bowden, Marian J Currie, Muareen Todkill, Mathias Schmidt, Sue Webeck, Rendry Del Rosario, Tim Bavinton, Alexandra Tyson

**Affiliations:** 1 Academic Unit of Internal Medicine, Australian National University Medical School, Canberra Hospital, PO Box 11, Woden, ACT, 2606, Australia; 2 Canberra Sexual Health Centre, Canberra Hospital, PO Box 11, Woden, ACT, 2606, Australia; 3 Sexual Health and Family Planning ACT 28 University, Avenue Canberra, Canberra, ACT, 2601, Australia

## Abstract

**Background:**

Opportunistic screening for chlamydia in non-clinical settings is becoming more common, but little is known about which settings (or events) offer the best return on investment. We measured the relative efficiency of each screening site and event during the conduct of a chlamydia education and screening outreach program which used a cash incentive to encourage participation (SOC2).

**Methods:**

SOC2 staff identified sites and organised events in non-clinical sites where young people were likely to congregate. 16 to 30 years olds were offered chlamydia education and a cash reward of AUD10 if they chose to be screened for chlamydia. Data collected during these activities were used to calculated five measures of efficiency: i) screening yield’ (proportion of people providing a sample), ii) proportion of positive tests, iii) ‘event screening tempo’ (number of screens performed for every hour that screening is offered), iv) ‘staff hour screening tempo’ (number of screens performed per hour of staff time) and v) ‘chlamydia detection tempo’ (number of positive tests detected per hour of screening).

**Results:**

3011 people (71% male) were screened during 18 events at 10 venues. Overall ‘screening yield’ was 43.8% (range: 20–77%) and 1.7% (95% CI: 1.1–3.0) of tests were positive (by event range 1–3%). Overall, the ‘event screening tempo’ was 23.7 screens per event hour (range 8.0–79.0), the ‘staff hour screening tempo’ was 6.5 screens per staff hour and the ‘chlamydia detection tempo’ was 0.4 positive tests per hour (range: 0–1.75).

**Conclusion:**

Assessing the efficiency of screening sites and programs should be integral to their conduct. We suggest the use of five measures to enable pragmatic assessment of any screening program. We introduce the terms ‘event screening tempo’, ‘staff hour screening tempo’ and ‘chlamydia detection tempo’ to describe three of these simple measures.

## Background

In Australia most chlamydia testing is performed in general practice and the amount of testing that occurs (over 500,000 tests per year) [1], in effect, constitutes a de-facto opportunistic screening program. It has been proposed that further reductions in prevalence can only be achieved by a co-ordinated screening program [2]. Such a program needs to target sexually active young people, as the majority of chlamydia infections occur in people aged 15 to 30 years, especially males who are less likely than females to engage with the formal health care system [3]. One way to reach this group is to offer screening in non-clinical settings.

During the initial phase of the ‘Stamp Out Chlamydia’ program (SOC) in the Australian Capital Territory (ACT), we pioneered the use of a small cash reward to encourage participation in chlamydia screening on the campuses of tertiary education institutions. We have shown that this novel approach is an effective and efficient means of screening large numbers of people for chlamydia in a short period with the added advantage of reaching a higher proportion of men than other opportunistic screening methods [4].

Public health initiatives, such as outreach chlamydia screening, need to be efficient to allow the best use of often scarce resources. Moreover, the Australian Health Ministers’ Advisory Council advise that program managers identify measurable indicators and collect appropriate data to monitor the success of any screening program [5].

Publications about programs such as national cervical and breast cancer screening present mortality and morbidity data as well as measures such as the number of cases averted, number needed to screen and classification statistics (sensitivity, specificity, positive predictive value, area under the Receiver Operating Curve) [6,7], but few authors have reported on the relative efficiency of different non-clinical screening venues, particularly those used to screen for chlamydia and other sexually transmissible infections (STI) and blood borne viruses (BBV). In one recent paper, researchers in the United States compared the labour costs associated with chlamydia screening in various non-clinical sites in two counties [8], while in the United Kingdom, a report on the piloting of fasTest HIV testing compared the efficiency of each community pilot site by reporting the number of sessions and hours of service delivered in each site and the total numbers of attendees and numbers of tests conducted [9].

In 2009, local government funding allowed the SOC program to continue in the ACT and chlamydia screening was offered in a wider range of non-clinical settings (SOC2). The program was conducted by staff from Canberra Sexual Health Centre and Sexual Health and Family Planning ACT. We used data collected during the first eight months of this second phase to compare the efficiency of the screening sites and events. Such information could be used by program managers and funders in many jurisdictions to maximise the efficiency of chlamydia, and other, screening activities.

## Methods

### SOC Program and data collection

SOC2 staff engaged with a range of small and large venues to establish a calendar of events. The target population were males and females between 16 and 30 years although those older than 30 were not refused testing. Data collection methods were the same for all events: two to six staff (depending on the expected size of the potential screening population) established a stand to draw attention to the chlamydia screening activity and potential participants were made aware that a AUD10 cash reward would be given to them in exchange for a urine sample. Participants were provided with written information about chlamydia testing and follow-up methods. Demographic data and a contact phone number (almost exclusively a mobile phone) were collected for each participant.

Individuals were considered to have been ‘exposed’ to the screening opportunity if they were given oral and/or written materials about chlamydia. This number was manually recorded using a hand tally counter and constitutes the estimate of the denominator population. Due to the nature of the events we were unable to determine whether people approached the staff more than once and were unable to rule out that some people were counted more than once in the denominator. However, before sending the specimens to the laboratory for testing we used probabilistic linkage methods (matching sex, name, birth date, and phone number - usually mobile number) to determine whether an individual had provided more than one specimen. Urine samples were tested for chlamydia using the Roche Cobas 4800 CT/NG Test, 2010. Those testing positive were contacted by the SOC2 staff and arrangements for treatment made through the Canberra Sexual Health Centre. Staff kept a log of the number of staff attending each event and the number of hours they worked directly with young people.

### Relative efficiency

Approval for a retrospective analysis of the data collected between October 2009 and June 2010 was obtained from the ACT Health Human Research Ethics Committee (ETHLR-11.226). To determine the efficiency of the chlamydia screening events and sites (tertiary campuses vs. other sites) we used the following measures: screening yield (total number of urine samples received), the positivity rate, the ‘event screening tempo’ (the number of screens performed for every hour that screening is offered to a target population), the ‘staff hour screening tempo’ (number of screens performed for every hour of staff time) and the ‘chlamydia detection tempo’ (the number of positive chlamydia tests detected per hour of screening).

## Results

During the analysis period, 18 events were conducted at 10 separate sites, 11 at tertiary campuses, three at a health club and one each at a motorsports event, a football club and an Aboriginal youth centre. By site and event results including testing and positivity rates and efficiency measures are presented in Table 1. The total denominator population was estimated to be 3011, ranging from 1000 (at the motorsports festival) to 40 (at one of the three health club events). 1318 individuals provided a urine sample, 929 (71.5%) males and 379 (29%) females, with sex not documented for 10 participants. 1077 (81.7%) of those tested were within the target age range of 16 to 30 years (range 13 to 63 years). The overall screening yield was 43.8% (1318/3011; range 20 to 77%). There were 22 positive chlamydia tests (15 in males and 7 in females, 20 in the 16 to 30 years age range and the other two in males aged 41 and 50 respectively) giving an overall positivity rate of 1.7% (95% CI: 1.1–2.5) with a range among the 18 events of 1.5% to 5.6%.

**Table 1:** Screening at Stamp Out Chlamydia activities in the ACT - October 2009 to July 2010

**Site**		**Staff**		**Screening**			**Chlamydia Screening tempo**		**Positive tests**	
	**Hrs screening offered**	**No.**	**Total hrs**	**No. exposed**	**No. screened** (% male)	*Yield %	^**#**^**Event**	^**†**^**Staff**	**No. (%,95% CI)**	^**&**^**Chlamydia detection tempo**
**Campus A**										
Event 1	3	5	15	136	98 (81.6)	72	32.7	6.5	2 (2%; 0–7.2)	0.67
Event 2	3	4	12	100	65 (98.5)	65	21.7	5.4	2 (3.1%;0–10.7)	0.67
Event 3	4	3	12	75	54 (100)	72	13.5	4.5	3 (5.6%;1.0-15.4)	0.75
Event 4	4	4	16	100	62 (100)	62	15.5	3.9	1 (1.6%;0–8.7)	0.25
**Campus B**	2	2	4	200	39 (61.5)	20	19.5	9.8	0	0
**Campus C**										
Event 1	3	3	9	200	69 (68)	35	23	7.7	1 (1.5%;0–7.8)	0.33
Event 2	3	3	9	100	47 (26:20)	47	15.7	5.2	0	0
**Campus D**										
Event 1	3	4	12	250	95 (55.3)	38	31.7	7.9	3 (3.2%;1.0-9.0)	1.0
Event 2	3	4	12	100	49 (0)	49	16.3	4.1	0	0
**Campus E**										
Event 1	3	4	12	100	37 (35.1)	37	12.3	3.1	0	0
Event 2	3	4	12	150	68 (33.8)	45	22.7	5.7	1(1.5%;0–7.9)	0.33
**Campus F**	2.5	3	7.5	200	126 (54.0)		50.4	16.8	2 (1.6%;0–5.6)	0.8
**Health club**										
Event 1	3	3	9	70	43 (18.6)	61	14.3	4.8	0	0
Event 2	3	3	9	70	47 (80.8)	67	15.6	5.2	0	0
Event 3	2	3	6	40	17 (41.2)	43	8.5	2.8	0	0
**Aboriginal Youth Centre**	4	3	12	50	32 (62.5)	64	8	2.7	0	0
**Football club**	3	3	9	70	54 (83.3)	77	18	6.0	0	0
**Motorsports festival**	4	6	24	1000	316 (85.3)	32	79	13.2	7 (2.2%;1.0-4.5)	1.75
**Total**	**55.5**		**201.5**	**3011**	**1318 (71.5)**	**44%**	**23.7**	**6.5**	**1.7% (1.1-2.5)**	**0.4**

*screened/exposed #screens/hour of activity †screens/staff hour ^&^positive tests/hr of activity.

**Figure 1:**
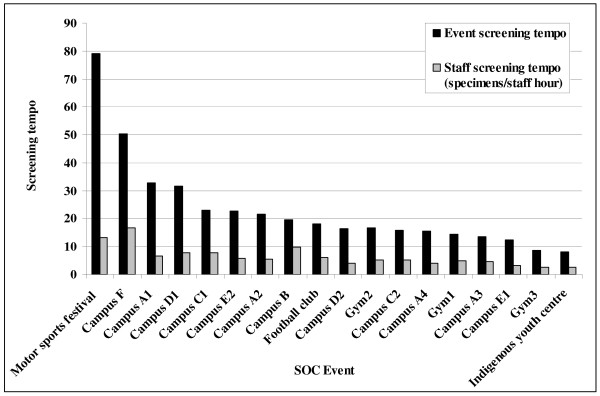
Comparison of event screening tempo with staff screening tempo by SOC event.

The 18 screening events were conducted over a total of 55.5 hours and required 211.5 staff hours (i.e. number of staff employed at each site multiplied by hours of the event). The overall ‘event screening tempo’ was 23.7 screens per event hour (range: 8–79) or one test every 2.5 minutes. The overall ‘staff hour screening tempo’ was 6.5 screens per staff hour (range: 2.7–16.8). The overall ‘chlamydia detection tempo’ was 0.4 positive tests per hour (range: 0–1.75). ‘Event screening tempo’ varied across the screening events (79 screens per screening hour i.e. one every 45 seconds for the motor sport s to 7.5 tests per screening hour i.e. one every eight minutes for the Aboriginal youth centre), while variations in ‘staff hour screening tempo’ were less marked (2.7 urine samples per hour of staff time for the Aboriginal youth centre to 16.8 urine samples per hour of staff time for one of the technology campuses). A comparison of these two measures for each site is presented in Figure 1.

The screening yield was higher at the tertiary campuses (809/1711, 69%) than the other venues combined (509/1300, 39.2%), although the highest screening yield was obtained at the football club.

## Discussion

Although establishing the relative efficiency of screening sites would appear to be an important component of the evaluation of any screening program, and of major interest to program managers and funding bodies, this is rarely reported. While studies of chlamydia screening activities routinely report the number and/or proportion of the target population that are screened, the time taken to perform this screening or the number of staff hours required is seldom documented. During the evaluation of the SOC2 program, we found that the use of five simple measures - number screened, cases identified, coupled with the newly labelled ‘event screening tempo’ (screens per event hour), the ‘staff hour screening tempo’ (screens per staff hour) and the ‘chlamydia detection tempo’ (positive tests per event hour) allowed timely and pragmatic assessment of the relative efficiency of 18 chlamydia screening events conducted in 10 non-clinical screening venues.

More complex assessment of the relative efficiency of different screening venues such as that performed by Morris et al. to compare chlamydia rates and the cost-effectiveness (labour time per sample collected and per case identified) among four types of venues (community outreach, schools, parenting centres and drug treatment/correctional facilities) using multivariate general linear models, provides rigorous and rich information.{Morris, 2010 #268} However, we suggest that the easily calculated measures we used are more accessible to a wider range of program providers. We also suggest that the three new terms we present here convey more information than simply presenting numbers screened, numbers positive, total staff hours and average tests per session and average tests per staff hour as are presented by Weathburn et al. in their assessment of the introduction of rapid HIV testing in four clinics in the United Kingdom [9].

If the number of chlamydia screens performed is the principal outcome measure of a program, then the cash incentive model appears to be efficient with nearly 24 individuals screened per hour i.e. one every 2.5 minutes that the screening activities were running. In comparison, Canberra’s two sexual health clinics conducted 6,950 chlamydia tests in 2009, approximately 140 tests per week or 4 tests per hour of service.

If case finding effectiveness is the measure of efficiency the SOC2 program overall was less efficient (0.4 cases per hour) than the non-clinical sites in California described by Morris et al. where the number of chlamydia cases per paid person-hour ranged from 2.0 (95% CI 1.0–4.2) in schools to 3.2 (95% CI 1.6–6.6) in parenting centres [8].

Case yields from population screening are usually lower than from clinic based services. The two local sexual health clinics reported positivity rates of around 5% compared to the SOC2 outreach program rate of 1.7%. In England in 2010, chlamydia positivity rates per 100,000 population reported from Genitourinary Medicine clinics and the National Chlamydia Screening Programme were 175.8 and 144.9 respectively {HIV and Sexually Transmitted Infections Department, 2011 #275} As is the case for clinic based screening, the case yield from outreach screening activities is increased through contact tracing with as many as 40% of sexual partners of index cases testing positive [10].

The ‘event screening tempo’ varied between different settings: we were able to collect 79 samples per hour (i.e. one every 45 seconds) at an annual motorsports festival, a population that many would consider to be ‘hard to reach’ under normal circumstances. A similar rate of just over 50 screens per hour was achieved at a university trivia night. The tempo of testing was slower at events where chlamydia screening was not the focal point and the potential participants were merely passing by, but even at the event with the slowest tempo we collected one test every eight minutes.

Comparisons of the ‘event screening tempo’ can be a little misleading. For example, the lowest screening tempo – eight screens per hour - was achieved at an Aboriginal Youth Centre, but the potential population to be screened at this event was only 50 individuals. By the end of the three hour event 64% of the group had been screened. On the other hand, the events with higher screening tempos may only reach small proportions of the potential population – around 30% in the case of the motorsports event.

Individual events require different numbers of staff. SOC activities involved a minimum of two and as many as six staff – the larger the event, the more staff required to deal with the anticipated number of urine samples. To control for this variation we also calculated the ‘staff hour screening tempo’ (the number of screens performed per hour of staff time allocated to the event). The narrower range of ‘staff hour screening tempo’ estimates compared to the ‘event screening tempo’ estimates for the different events suggests that the former may be a more useful calculation of relative efficiencies (Figure 1). It is important to note that we calculated the ‘staff hour screening tempo’ using only the hours staff were engaged face to face with participants not the time required to prepare for outreach screening events, so comparisons with Morris et al. who did include both paid and volunteer staff hours cannot be made. These data will be collected for future events.

While the event and staff hour screening tempo can be accurately determined from our data, the screening yield (i.e. number tested/number exposed to the activity) is harder to calculate and is subject to considerable error. Calculating the number of individuals attending a closed event (such as Tertiary Campus F which was a trivia night or Campus B which was an international student’s meeting) is relatively easy, but the estimated number of people who have been made aware of SOC screening at a larger event has a reasonably wide confidence bound. We therefore recommend caution in comparing the screening yields of the activities reported here.

Of course, decisions about where to locate chlamydia screening sites and events should also be guided by knowledge of the local epidemiology of the infection. Also, while it may be inefficient to screen some populations, issues of access and equity also need to be considered. The proportion of positive chlamydia tests in our screened population was 1.7%; the confidence intervals are wide for the individual activities which showed higher proportions. The aggregate ‘chlamydia detection tempo’ was 0.4 positive tests per hour of screening. In other words, one positive test was detected for every 2.5 hours of screening. Obviously this rate is determined by the prevalence of chlamydia in the target population and the event related screening tempo: at the motorsports event, where the prevalence of chlamydia was 2.2%, the detection tempo was 1.75 positive tests per hour (i.e. a positive test for every 34 minutes of screening). It is also interesting to note that two of seven positive tests detected at this event came from men considerably older than the target age group. In populations where the prevalence of chlamydia is higher than was observed in our study, the chlamydia detection tempo will also be higher.

## Conclusion

Although the original SOC study demonstrated the relative benefit of cash incentives in increasing the tempo of screening, we are unable to find any other published Australian studies that calculate the screening and detection tempo estimates for a chlamydia screening program. We suggest that a pragmatic assessment, using simple measures of efficiency, should be integral to the conduct of screening programs, and that such evaluations should be planned from the outset so appropriate data are collected. We have introduced three new terms to describe three measures of screening efficiency: the ‘event screening tempo’ the ‘staff hour screening tempo’ and the ‘chlamydia detection tempo’. These methods can be used, in combination with the more commonly used proportion of positive tests and the screening yield, to provide quick and simple estimates of the efficiency of any screening program. Furthermore, we believe that there is a need for a randomised study to compare the acceptability, screening tempo, screening yield and positive test yield of this approach in a variety of community settings beyond the ACT.

## Competing interests

The authors declare that they have no competing interests.

## Authors’ contributions

FJB was the PI and was the lead author for this paper. MJC conducted the data analysis. MT is the project officer for the SOC2 Project, led the data collection and assisted with data analysis, MS, SW, TB and AT formed the project team and collected the data. All authors contributed to study design and the preparation of the manuscript. All authors read and approved the final manuscript.

## Pre-publication history

The pre-publication history for this paper can be accessed here:

http://www.biomedcentral.com/1471-2458/12/341/prepub
